# Suit against the American Medical Association for Damages

**Published:** 1909-07

**Authors:** 


### EDITORIAL DEPARTMENT.

This issue begins the 25th year of the TEXAS MEDICAL JOURNAL, under one—the same—and original management. I salute my readers, make my best bow and return thanks for cordial, long sustained and enthusiastic support. It has been called the "Rock-ribbed, Time-tried and Fire-tested Old Red Back." It enters upon its 25th year with all the vim, vigor, courage and determination to stand for the rights and interests of the regular profession that characterized its earlier years, abating not a jot or tittle of its antagonism towards mixing with the pathies, quacks and negroes, notwithstanding the unfortunate attitude in which our State Association has been placed by those in control, a new element, who seemingly are willing to the sacrifice—to please the powers at Chicago. I call attention to the steady advertising support accorded the "Red Back" notwithstanding the machinations of those who would gladly silence its Gatlin guns, and the lying statement that it is controlled by a proprietary association. I declare on honor, and may some day prove in court that, with a single exception, not one of my advertisers is a member of any such association.

Here's a smile for those who love me,
And a kick for those who hate;
Whatever skies above me,
Here's a heart for any fate.

### Suit Against the American Medical Association for Damages.

Two separate suits for $100,000 each for damages have been brought in the United States Circuit Court in Chicago, by Dr. S. Lewis Summers and the Organic Chemical Company of Philadelphia against the American Medical Association for alleged willful misrepresentations about the products of the plaintiff company, published in the *Journal of the American Medical Association.* Dr. Summers claims that the report of the Council of Pharmacy touching certain products of the company, made by Torrald Sollman, of the Council, and published in the *Journal* of September 5, 1908, and in May 8, 1909, contains libelous statements. He claims that he is prepared to show by such eminent chemists as Prof. Samuel P. Sadtler, Ph. D., LL. D., President of the College of

Pharmacy, and Henry Beates, Jr., M. D., President of the Pennsylvania Medical Examining Board, that Dr. Sollman's "findings," upon analysis of Iodomuth, for instance, upon which finding and report said Iodomuth was refused recognition, but was denounced in print, were incorrect, false, in fact; hence the damage to their business.

The issue of this suit will be watched with interest by the medical profession. It will be a test case whether a self-constituted censorship, having no shadow of authority in law, can denounce as fraudulent or dishonest any pharmacal preparation upon the findings of a chemist with whom other chemists may and do differ, and thus destroy a manufacturer's business. It certainly looks high-handed. The wonder is that no such suit has been brought before. The suit promises to be most sensational, inasmuch as the plaintiffs will seek to show, and they declare they will "expose a systematic attempt by the Council to give a monopoly to certain German chemical manufacturers, shutting out American producers."

It will be observed that the suit is against the American Medical Association, a rich corporation, chartered, and not against Dr. Sollman, or the Council, or the trustees of the *Journal*. Every individual member of the A. M. A. is responsible at law for the utterances of its official organ. And the same is true of the several State Medical Associations and their journals, especially when, by resolution or otherwise, the House of Delegates specifically endorses the management and approves of the editor's libelous statements. It will not do to stake one's pile on the infallibility of a chemist, or on the uncertain temper of a fledgling editor.

